# Residential Proximity to Freeways is Associated with Uncontrolled Asthma in Inner-City Hispanic Children and Adolescents

**DOI:** 10.1155/2010/157249

**Published:** 2010-06-13

**Authors:** Peter Huynh, Muhammad T. Salam, Tricia Morphew, Kenny Y. C. Kwong, Lyne Scott

**Affiliations:** ^1^Division of Allergy and Immunology, Department of Pediatrics, Los Angeles County and University of Southern California Medical Center, University of Southern California Keck School of Medicine, Los Angeles, CA 90033, USA; ^2^Department of Preventive Medicine, University of Southern California Keck School of Medicine, Los Angeles, CA 90033, USA; ^3^Southern California Chapter, Asthma and Allergy Foundation of America, Los Angeles, CA 90036, USA; ^4^Division of Allergy-Immunology, Department of Pediatrics, Harbor-UCLA Medical Center, University of California, Los Angeles, CA 90095, USA

## Abstract

*Background*. Proximity to heavy traffic has been linked to increased asthma severity. However, it is unknown whether exposure to heavy traffic is associated with the ability to maintain asthma control. *Objectives*. This study examines whether exposure to heavy traffic is associated with the ability to maintain asthma control in inner-city children. *Methods*. 756 inner-city asthmatic Hispanic children were followed for one year in a pediatric asthma management program (Breathmobile). At each scheduled visit, asthma specialist tracked patients' asthma severity and managed their asthma based on the NAEPP guidelines. The patients' residential distance from the nearest freeway was calculated based on residential address at study entry. Distance to nearest freeway was used as a surrogate marker for high exposure from traffic-related air pollutants. *Results*. Patients who lived near a freeway were significantly more likely to have asthma that was not well controlled (*P* = .03). Patients with intermittent and mild baseline severity have a two-fold increased risk of having asthma that is uncontrolled if they lived <2 miles from a freeway (OR = 2.2, *P* = .04). *Conclusion*. In children with asthma, residential proximity to freeways is associated with uncontrolled asthma.

## 1. Introduction

Increasing evidence suggests that residential proximity to traffic sources increases the risk for the development of asthma [1] and asthma morbidity [2]. In recent studies, air pollution from heavy road traffic has been linked to increased asthma exacerbations, and asthma-related emergency department visits and hospitalizations [3, 4]. A major source of air pollution in urban areas is particulate matter from diesel exhaust [5]. Asthmatic children who live near freeways are susceptible to diesel exhaust [6, 7]. Particulate matter produces a strong inflammatory response in the airways involving various cells, mediators, cytokines, and adhesion molecules [8]. This is believed to contribute to worsening asthma severity. Particulate matter exposure has been associated with decreased lung function, increased symptoms of respiratory distress, increased use of asthma medications, and increased emergency room visits and hospitalizations [9–11]. 

The 2007 National Asthma Education and Prevention Program (NAEPP) guidelines emphasize the importance of asthma control [12]. Treatment towards asthma control reduces impairment and risk. While the majority of asthmatic patients can achieve and maintain asthma control, a significant number never achieve control despite guideline-based therapy [13]. Factors contributing to poor asthma control include comorbid conditions such as upper airway disease [14], active smoking [15], and obesity [16]. Environmental factors and variants in genes in the oxidant stress pathway are also associated with increased asthma risk [17]. Although residential traffic-related exposures have been associated with asthma severity, whether these factors affect asthma control in children remains largely unknown. The aim of this study is to evaluate the impact of residential proximity to a major freeway on the differential ability to maintain asthma control.

## 2. Methods

### 2.1. Inclusion Criteria

Our study population consisted of Hispanic children enrolled in an asthma specific disease management program (Breathmobile) at Los Angeles County and University of Southern California Medical Center. Details of the program have been described earlier [18, 19]. Patients were enrolled in this study from June 1998 to December 2006. Patient care is based on 1997 National Asthma Education and Prevention Program (NAEPP) guidelines [20]. Patients who met all the following criteria were enrolled: (1) diagnosis of asthma, (2) 3–18 years of age, (3) enrollment in program ≥365 days, (4) ≥3 follow-up visits during the first year, (5) ≥ one visit during the first half of the second year (366–548 days post entry), (6) ≥ one visit during the second half of the second year (549–730 days post entry), (7) asthma symptoms (cough, wheeze, chest tightness, shortness of breath) and frequency occurrence (daytime/nighttime) recorded in electronic medical record (Asmatrax) at each visit, (8) baseline asthma severity recorded in Asmatrax, (9) asthma specialist assessment of asthma control recorded in Asmatrax for all visits and (10) asthma specialist-rated adherence to the treatment plan. Patients who did not fulfill all inclusion criteria or were nonadherent to the treatment plan were excluded. Patient consent was not required as this is a retrospective analysis of clinical data. This study was reviewed and approved by the Institutional Review Board at LAC+USC Medical Center.

### 2.2. Measures and Data Collection

Using 1997 NAEPP guidelines, baseline asthma severity was assessed and recorded at each initial visit by asthma specialist. Asthma specialists consist of board eligible/board-certified allergist/immunologist. Asthma control was determined and recorded at each follow-up visit. The asthma specialist rated the patient's asthma as *controlled* if all of the following criteria were met: (1) symptoms (cough, wheeze, chest tightness, shortness of breath) ≤2 days per week and ≤2 nights per month for the 4-week period before the current visit, (2) no asthma exacerbations in the preceding visit interval (defined as no rescue use of systemic steroids, ED visits, or hospitalizations), (3) FEV1 >80% of predicted or >80% of patient's personal best (pulmonary function studies performed with a MultiSpiro SX Spiro meter San Clemente, Calif based on recommendations from American Thoracic Society guidelines), and (4) no reported limitations on a patient's activities or exercise caused by asthma. 

The asthma specialist also recorded an estimate of adherence with the management plan since the last visit (compliant, noncompliant, or off therapy), as well as changes to the prescribed treatment plan (no change, step-up, step-down, restart, or stop). Patients are considered to be adherent when the asthma specialist perceives that they are using their controller medications on an undefined regular basis. A rating of noncompliant or off therapy might be due to patient/family behavior, such as choosing to use controller medications episodically (noncompliant) or not using them at all (off therapy), or the patient might be off therapy because of socioeconomic factors beyond the family's control (e.g., inability to pay for medications, problems in getting prescriptions filled, inability to get prescriptions refilled, and cancellation of Medi-Cal coverage). 

Treatment was based upon the 1997 NAEPP guidelines although the type and dose of controller and reliever medications were left to the discretion of the individual asthma specialist provider. Patients had unrestricted access to inhaled corticosteroids (ICS), long acting *β*2 agonists (LABA), leukotriene receptor antagonists (LTRA), and short acting *β*2 agonists (SABA). 

Patients were stratified into two different asthma control groups. *Uncontrolled *asthma was defined as control recorded at <80% of follow-up visits. *Well-controlled* asthma was defined as control recorded at ≥80% of follow-up visits. The 80% threshold for *well-controlled* asthma is based on a previously published study that applied the criteria as an indicator of well-controlled disease prior to the 2007 NAEPP Guidelines [21].

The patient's residential distance from the nearest freeway was calculated based on the residential address at study entry. Residential address was geocoded using Geocoder online software (http://geocoder.us). Webtonix online software (http://www.webtonix.com/maps/) was used to calculate the distance from each residence to the nearest freeway, defined as an interstate freeway, US highway, or restricted access highway. 

### 2.3. Outcome Measures

The primary outcome of this study is the relationship between proximity to freeways and asthma control. Secondary outcome include interaction between baseline asthma severity, asthma control, and proximity to freeways. 

### 2.4. Statistical Analysis

**Figure 1:**
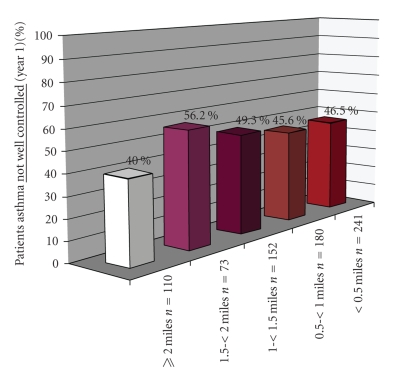
Distance from freeway described in relation to asthma Control during year one participation in Breathmobile program, stratified by proximity to freeway.

Distributional differences in patient characteristics including age, gender, disease severity, baseline morbidity, and residential proximity to freeway by asthma control status during year one (well controlled versus not well controlled) assessed for significance by independent *t*-test (continuous factors) and chi-square test (categorical factors) (Table 1). Bar chart created to further describe percent of patients whose asthma not well controlled during year one in relation to proximity to freeway within each disease severity category (Figure 1). Logistic regression analysis provided corresponding odds ratios indicating likelihood asthma not well controlled by distance to freeway (<2 miles versus ≥2 miles) and potential confounding factors within severity strata (Table 2). Crude assessment examining lower threshold values (< .5 miles,  .5–< 1 mile, 1–< 1.5 miles, 1.5–< 2 miles, ≥2 miles) showed no appreciability between group difference in likelihood asthma not well controlled until residence at two or more miles from freeway. Investigation extended to determine whether adjustment was required in examining relationship between freeway proximity (<2 versus ≥2 miles) and asthma control. Backward selection procedure with “distance” forced into model within each severity strata (intermittent-mild and moderate-severe). Potential confounding factors investigated in adjusted analyses: age (3–5 years, 6–18 years), gender (male, female), # freeways within proximity of home (<2, ≥2), baseline morbidity (pre year): asthma attacks (<2, ≥2), ED visits (none, any), hospitalizations (none, any), and school absenteeism due to respiratory symptoms (<5, >5 d). Analyses conducted using SPSS version 12.0 software (SPSS Inc, Chicago, IL). 

## 3. Results

### 3.1. Study Population

The general patient characteristics are described in Table 1. The mean age of patients is 8.5 years (SD, 3.2, years) at time of enrollment. 59% of patient population is male. All patients enrolled in this study are Hispanic. Patients included all baseline severity classifications. The residential distance from the nearest freeway ranged from 0.023 to 3.78 miles (mean, 1.05 miles; SD, 0.83 miles). Very few patients in our study population lived more than 3 miles from a freeway (3.6%). 53% of our patients had well-controlled asthma (≥80% control on follow-up visits). Factors contributing to patients having uncontrolled asthma include baseline severity (*P*  <  .01) and residential proximity to freeway (*P*  =  .03). 

### 3.2. Asthma Control and Proximity to Freeways

Residential proximity to a freeway is significantly related to asthma control (*P*  =  .03) (Table 1). Patients who lived near a freeway were significantly more likely to have asthma that was not well controlled. The asthma control group differences (uncontrolled versus well-controlled) became most apparent the closer a patient resides from a freeway. 

### 3.3. Asthma Control and Baseline Asthma Severity

Asthma control is significantly related to baseline asthma severity (*P*  <  .01) (Table 1). Patients with uncontrolled asthma have more moderate to severe baseline severity than patients whose asthma was well controlled (61% versus 47%, resp.). A more pronounced difference was noted for severe persistent disease (31.6% versus 19.2%) compared to moderate persistent disease (29.7% versus 27.4%), with respect to uncontrolled versus well-controlled asthma. Measures of baseline asthma morbidity such as the frequency of asthma attacks, emergency room visits, and hospitalizations did not significantly impact the patient's level of asthma control. However, school absenteeism (≥5 days) was significantly associated with uncontrolled asthma (*P*  =  .03) for all asthma baseline severity groups. 

### 3.4. Proximity to Freeways, Baseline Asthma Severity, and Asthma Control

Baseline asthma severity interacted in the relationship between proximity to freeway and asthma control. Figure 1 shows percent of patients with uncontrolled asthma in relation to proximity to freeway and baseline disease severity. Patients with intermittent and mild baseline severity have a two-fold increased risk of having asthma that is uncontrolled if they lived <2 miles from a freeway (OR  =  2.2, *P*  =  .04). For patients with moderate to severe baseline severity, there is a 20% increased risk of having asthma that is not controlled if they lived <2 miles from a freeway, although this did not reach statistical significance (OR  =  1.2, *P*  =  .5). Patient factors including age, gender, and baseline morbidity measures did not confound nor modify the observed relationship between proximity to freeway and asthma control in either baseline severity group. Substantial school absenteeism during year prior to program entry (≥5 days) did correspond to increased likelihood of uncontrolled asthma (<80% control on follow-up visits) in moderate to severe patients (*P*  =  .03).

## 4. Discussion

Results of this study provide strong evidence that residential proximity to freeway is associated with uncontrolled asthma. For all baseline asthma severity groups, patients who lived close to a freeway (<2 miles) were more likely to have uncontrolled asthma. Proximity to freeways played a significant role in uncontrolled asthma despite regularly scheduled visits and guideline-based care by asthma specialists. The 2007 NAEPP guidelines recently emphasize the importance of asthma control. However, not all patients achieve control despite guideline-based care [13, 18]. Exposure to heavy traffic densities may play a key role in the subset of patients who do not achieve asthma control despite regularly scheduled patient care and structured assessment.

Asthma control was associated with underlying baseline asthma severity. Interestingly, patients with baseline severe persistent asthma lived further from a freeway compared to subjects with milder phenotypes. There may be several reasons for this. Patients with more severe disease may move away from freeways, leaving a greater relative percentage of patients with milder disease living in close proximity to freeways, a so-called “survivor” effect. Patients with worse severity may have multiple asthma triggers besides exposure to traffic pollution. Their asthma control may be complicated by a varied physiological response to medications, genotype-associated response patterns, and other social and environmental factors that influence disease activity. Alternatively, patients with milder baseline severity may be able to tolerate higher exposures to traffic densities. 

**Figure 2:**
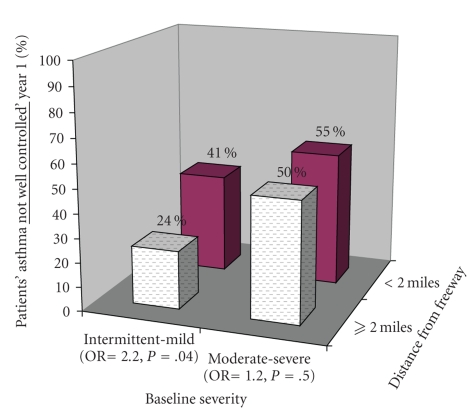
Distance from freeway versus asthma control during year one participation in Breathmobile program, stratified by baseline disease severity.

Baseline asthma severity interacted with the relationship between proximity to freeway and asthma control. As shown in Figure 2, the effect of proximity to freeway on maintenance of asthma control was greater in patients with intermittent to mild persistent baseline severity than patients with moderate to severe baseline severity. Proximity to freeways may have an impact on certain asthma phenotypes. Patients with intermittent to mild persistent severity may demonstrate exaggerated hyperresponsiveness to particulate matter and may have less sensitization to other environmental triggers. Patients with more severe asthma phenotypes may have more pronounced triggers for their asthma and the single effect of proximity to freeways may be minimized in the context of multiple exacerbating factors. 

In our primarily treated population, baseline morbidity (number of asthma attacks, emergency room visits, and hospitalizations) did not appreciably impact patient's level of asthma control, with the exception of school absenteeism. Although patients reporting one or more hospitalizations per year appeared to have an increased likelihood of uncontrolled asthma during year one, the small percentage of patients reporting this morbidity event per year (7.5%) may have limited the power to detect a significant observed increased risk. As expected, substantial school absenteeism did correspond to an increased likelihood of uncontrolled asthma (<80% control on follow-up visits) in moderate to severe patients (*P*  =  .03).

We recognize some limitations to our study. First, this was a retrospective observational study and analysis was performed among patients in a specialized treatment program where patient adherence with prescribed treatment plan is relatively high (~>70%). This may have minimized confounding effects related to treatment adherence. Nonadherence has been associated with poor asthma control [22]. Second, patients enrolled in this study were primarily Hispanic children living in the inner city. The results of this study may not be universally applicable to more ethnically and socioeconomically diverse communities. The insurance distribution indicated that the study population was predominately from low socioeconomic status. The majority of our patients qualified for public funded health insurance such as MediCal (58%), 29% had no insurance, and only 13% had private insurance. Due to the relatively small percentage of patients with private insurance (applied as a proxy for SES), the ability to investigate SES as potential confounder was limited. Future studies that include a larger percentage of patients from higher SES groups would enable more thorough investigation of socioeconomic status as a potential confounding factor. Finally, patients were enrolled into our study at different points in time. Patterns of asthma control are quite variable despite long-term guideline-based care [19]. Patients may develop worsening of their asthma caused by seasonal fluctuation, however, this effect was minimized by examining control over the course of a year. 

Although there are some limitations to our study, the number of patients and the length of follow-up are major strengths in our study. Both of these factors minimize the seasonal impact on asthma control and the effects of the season of entry into the program on asthma control. Another major strength of our study was the structured evaluation of patients at each visit based upon NAEPP guidelines. This approach allows for systematic data collection to track asthma control in relation to exposure to heavy traffic. Previous studies have investigated the link between heavy traffic and asthma severity [3, 23]. To our knowledge, this is the first study that has demonstrated an association between traffic exposure and asthma control. 

 This study adds to the mounting evidence of adverse effects of traffic-related air pollutants on asthma. Not only does traffic related air pollution increase the risk for asthma development, asthma morbidity, and reductions in lung growth in children [1, 2, 24], it plays a significant role in the ability to control asthma despite regularly scheduled patient care in an asthma disease specific management program providing guideline-based care. Given the magnitude of the problem, strategies for reducing traffic-related pollutants are needed to decrease the burden of asthma on children. Furthermore, clinicians should consider evaluating traffic-related exposures in patients with poor asthma control.

## Figures and Tables

**Table 1:** Characteristics of patients described by asthma control during year one participation in Breathmobile program.

		Asthma control during year 1^(a)^		
	Overall *N* = 756	Well controlled *N* = 402	Not well controlled *N* = 354	*P*-value^(a)^
Age (years; mean [SD])	8.5 yrs [3.2]	8.3 yrs [3.1]	8.7 yrs [3.3]	*P* = .13
Female sex	40.6%	40.4%	40.8%	*P* = .91
Baseline Severity:				
Intermittent	21.7%	26.4%	16.4%	*P* < .01
Mild persistent	24.9%	27.1%	22.3%	
Moderate persistent	28.4%	27.4%	29.7%	
Severe persistent	25.0%	19.2%	31.6%	
Baseline morbidity (past year):				
Asthma Attacks (two or more)	14.9%	16.4%	13.3%	*P* = .23
ED visits (one or more)	26.7%	26.4%	27.1%	*P* = .82
Hospitalizations (one or more)	7.5%	6.2%	9.0%	*P* = .14
School absenteeism (≥5 d)	22.1%	18.9%	25.7%	*P* = .03

Distance from freeway^(c)^:				
Mean miles [SD]	1.0 mi [0.83]	1.1 [0.88]	1.0 mi [0.76]	*P* = .29
Range	(0.02–3.78)	(0.02–3.78)	(0.02–3.51)	
<1 mile	55.7%	56.5%	54.8%	*P* = .03
1-<2 miles	29.8%	27.1%	32.8%	
2-<3 miles	11.0%	11.2%	10.7%	
≥3 miles	3.6%	5.2%	1.7%	

^(a)^Patient's asthma control during year 1 Breathmobile program participation: well controlled (asthma control maintained at 80% or more of follow-up visits during year 1) and not well controlled (asthma control maintained at fewer than 80% of follow-up visits during year 1). The majority of patients in this category (68%) maintained asthma control at 50%–79.9% of follow-up visits).

^(b)^ *P*-value: Significance asthma control group differences in distributions based on independent *t*-test (continuous factors) and chi-square test (categorical factors).

^(c)^Point of reference: Patient's home.

**Table 2:** Influence of distance from freeway and patient characteristics on asthma control during year one participation in Breathmobile program, stratified by baseline asthma severity. OR: odds ratio.

	OR = Likelihood patient's asthma “not well controlled” during year 1 comparing patients in respective to reference category (—)			
	Intermittent-mild asthma *N* = 352		Moderate-Severe Asthma *N* = 404	
	% Asthma not well controlled	Unadjusted OR (95%CI)	% Asthma not well controlled	Unadjusted OR (95%CI)

Overall	39%	—	54%	1.8 (1.4,2.4)
Distance from freeway:				
<2 miles	41%	2.2 (1.1,4.7)^†^	55%	1.2 (0.7,2.0)
≥2 miles	24%	—	50%	—
Potential confounding factors:				
# Freeways				
Less than two freeways	38%	—	54%	—
Two or more freeways	43%	1.2 (0.8,2.0)	54%	1.0 (0.7,1.6)
Age (years)				
3–5 years	44%	1.3 (0.8,2.2)	49%	0.8 (0.5,1.3)
≥6 years	38%	—	55%	—
Gender:				
Female	39%	1.0 (0.7,1.6)	53%	1.0 (0.7,1.5)
Male	39%	—	54%	—
Baseline morbidity (past yr):				
Asthma Attacks:				
Less than two	40%	—	55%	—
Two or more	26%	0.5 (0.2,1.1)	49%	0.8 (0.5,1.3)
ED visits:				
None	39%	—	55%	—
One or more	41%	1.1 (0.6,1.9)	51%	0.9 (0.6,1.3)
Hospitalizations				
None	39%	—	53%	—
One or more	44%	1.2 (0.4,3.4)	61%	1.4 (0.7,2.7)
School absenteeism:				
Less than five days	39%	—	51%	—
Five or more days	41%	1.1 (0.6,1.9)	63%	1.7 (1.1,2.7) ^†^

*P*-value: ^†^ ≤ .05, ^‡^ ≤ .01 (based on logistic regression analysis).
